# Study on the Effects of Different Drying Methods on Nutritional Quality of *Dictyophora rubrovalvata* Soup Based on Metabolomics

**DOI:** 10.3390/foods15213930

**Published:** 2026-11-02

**Authors:** Jing Zhang, Junhui Wang, Li Gao, Lei Zhang, Yuping Zhang

**Affiliations:** 1 Sericultural Research Institute, Anhui Academy of Agricultural Sciences, Hefei 230061, China; 15357033780@163.com (J.Z.); zhanglei5900@126.com (L.Z.); 2 Engineering Research Center of Bio-Process, Ministry of Education, School of Food and Biological Engineering, Hefei University of Technology, Hefei 230601, China; gaoli@hfut.edu.cn; 3 Edible and Medicinal Mushroom Innovation Centre, Anhui Academy of Agricultural Sciences, Hefei 230001, China

**Keywords:** drying methods, *Dictyophora rubrovalvata* soup, metabolomics, taste profile, bioactivity

## Abstract

This study investigated the effects of different drying methods on the quality and metabolic characteristics of *Dictyophora rubrovalvata* soup. Soups were prepared using *D. rubrovalvata* treated by means of vacuum freeze-drying (FDS), hot-air drying (HADS), far-infrared drying (FIDS), and combined hot-air and far-infrared drying (HAFIDS). The soups, referred to as FDS, HADS, FIDS, and HAFIDS, respectively, were evaluated using an electronic tongue, UV spectroscopy, fluorescence spectroscopy, and in vitro antioxidant and hypoglycaemic activity assays. By combining these evaluations with non-targeted metabolomics techniques, this study analysed the physicochemical properties, biological activities, and patterns of change in non-volatile metabolites. The spectral results indicated that the Maillard reaction occurred in all groups of *D. rubrovalvata* soup during the cooking process, with the reaction being more pronounced in the heat-dried group. Among the four soup samples, HAFIDS exhibited superior DPPH radical scavenging, FRAP, and α-amylase and α-glucosidase inhibitory activities, along with the highest abundance of umami-related metabolites, revealing that combined hot-air and far-infrared drying achieves a favourable balance between flavour and bioactivity. Metabolomics identified a total of 716 metabolites, with 238 differentially expressed metabolites screened, primarily enriched in pathways such as amino acid metabolism, the tricarboxylic acid cycle, glucose metabolism, and nucleotide metabolism. Twelve key taste-related amino acids were identified, with significant differences observed between the different drying groups in terms of umami, sweet, and bitter amino acids, as well as organic acids and sugar alcohols. This study systematically investigated the metabolic changes underlying the gustatory quality and bioactivity of *D. rubrovalvata* soup through a metabolomics analysis of drying methods. It demonstrated that combined hot-air and far-infrared drying is more suitable for the high-value processing of *D. rubrovalvata* soup, providing a theoretical basis and data support for the flavour optimisation and processing of edible fungus soups.

## 1. Introduction

*Dictyophora rubrovalvata*, a species within the genus *Dictyophora* and the family Phallaceae, is a highly valuable fungus with both nutritional and medicinal value. Research indicates that *D. rubrovalvata* is rich in protein, minerals, and vitamins and contains abundant bioactive compounds, including flavonoids, polysaccharides, phenolic substances, and dietary fibre [1]. These active ingredients have demonstrated significant efficacy in terms of antioxidant activity, blood glucose regulation, and immune function modulation [2]. In this study, the term ‘nutritional quality’ refers to the overall profile of non-volatile, taste-active, and bioactive metabolites (free amino acids, sugars and sugar alcohols, organic acids, and nucleotides and their derivatives), together with the associated in vitro antioxidant and hypoglycaemic activities, rather than the conventional proximate nutritional composition.

*D. rubrovalvata* has a distinctive flavour and aroma and is often combined with other ingredients in soup preparation. The texture of these soups is influenced by non-volatile compounds found in *D. rubrovalvata*, such as amino acids, nucleotides, organic acids, and soluble sugars [3]. However, the flavour release patterns of dried *D. rubrovalvata* during processing, and the effect of different drying techniques on the resulting soup’s taste and aroma, remain poorly understood.

Drying is a crucial method in food processing and preservation; however, different drying techniques significantly affect the nutritional contents and flavour compounds of foodstuffs. As temperatures increase, complex chemical reactions occur. In addition to impacting flavour, these processes modify the physicochemical characteristics of *D. rubrovalvata* [4,5]. Y. Sun et al. employed four methods to prepare *Hypsizygus marmoreus* soup, revealing significant post-cooking increases in polysaccharide, polyphenol, and amino acid levels [6]. H. Yang et al. evaluated the effects of different drying methods on the physical properties and flavour of porcini mushroom soup [7]. They found that freeze-dried and heat-pump-dried porcini mushroom soup exhibited higher antioxidant, hypoglycaemic, and anti-adipogenic activities in vitro. Furthermore, FDS was abundant in pyrazines, furans, and organosulfur constituents, which enhanced umami, texture, and mouthfeel. Existing research has largely focused on the effects of drying on the conventional physicochemical properties, volatile flavour compounds, and nutritional components of edible fungi; however, the underlying mechanisms by which drying methods influence the formation of non-volatile flavour compounds and bioactive substances in *D. rubrovalvata* soup from a metabolic perspective remain unclear. There is currently a lack of systematic research into the intrinsic relationship between metabolic changes induced by drying processes and alterations in flavour characteristics and bioactivity.

This study used *D. rubrovalvata* as the test material and employed four drying methods. In combination with UV absorption spectroscopy, fluorescence spectroscopy, and in vitro assessments of antioxidant and hypoglycaemic activity, this study used UPLC-Q-Exactive Plus MS non-targeted metabolomics to systematically analyse the effects of different drying methods on the metabolite composition, key metabolic pathways, and core flavour compounds of *D. rubrovalvata* soup [8]. The findings provide the basic mechanisms by which drying methods regulate the gustatory quality and bioactivity of *D. rubrovalvata* soup through metabolic pathways, thereby providing a scientific basis for the high-value utilisation of *D. rubrovalvata*, soup flavour regulation, and the development of functional foods.

## 2. Materials and Methods

*D. rubrovalvata* (cultivar ‘Wansun 2’), sourced from Anhui Province, was dried and pulverised for storage and subsequent use. This cultivar is officially registered with the Anhui Provincial Registration Committee for Non-major Crop Varieties (Certificate No. 2112005), and the identity of the material was confirmed by morphological examination and internal transcribed spacer (ITS) sequencing (see Supplementary Materials). The *D. rubrovalvata* drying methods were as follows. Vacuum freeze-drying (FD): Fresh *D. rubrovalvata* was pre-frozen at −80 °C for 12 h and then freeze-dried in a freeze dryer with the cold trap at approximately −80 °C and the drying chamber at approximately −55 °C [9]. Combined hot-air and far-infrared drying (HAFID): *D. rubrovalvata* samples were placed in a drying oven at 60 °C for 2 h, then transferred to a far-infrared drying oven at 70 °C for 4 h. Hot-air drying (HAD): Samples were dried in an electric forced-air drying oven at 60 °C for 8 h with forced air circulation at the oven’s fixed setting [10]. Far-infrared drying (FID): Samples were dried in a far-infrared drying oven at 70 °C for 8 h with a rated power of 1400 W and a far-infrared wavelength range of 2–15 μm emitted from quartz tube radiators [11]. The dried *D. rubrovalvata* was then pulverised and sieved through a 100-mesh screen for later use. *D. rubrovalvata* soup with a 1:30 solid-to-liquid ratio was prepared from *D. rubrovalvata* dried via these four methods, heated at 70 °C, and boiled for 25 min; the resulting soups were named FDS, HAFIDS, HADS, and FIDS. After boiling, the soups were filtered. Portions of the four soups were used for metabolomics analysis, while the remainder underwent additional physicochemical experiments using a vacuum freeze dryer (FD-1A-50, Beijing Boyikang Laboratory Equipment Co., Ltd., Beijing, China).

### 2.1. Electronic Tongue Experiment

The method for using the electronic tongue (Electronic tongue (SA402B, Intelligent Sensor Technology, Inc., Atsugi, Japan)) was based on the relevant literature and modified accordingly [12,13].

### 2.2. Spectral Analysis

#### 2.2.1. Ultraviolet–Visible Spectral Analysis (UV–Vis)

Samples (1 mg/mL) were prepared using phosphate-buffered solution. Following the method from [14] with modifications, the absorbance values were measured using a UV spectrophotometer UV spectrophotometer (TU-1810, Persee, Beijing, China) across the wavelength range of 200 to 500 nm. The measurements were repeated three times for each sample.

#### 2.2.2. Fluorescence Spectroscopy Experiment

Following a method described in the literature, with modifications [15], the fluorescence spectral characteristics of various samples were determined using a fluorescence spectrophotometer (F97Pro, Lengguang, Shanghai, China). Samples were prepared at a concentration of 1 mg/mL in 10 mM phosphate buffer, with the solution pH adjusted to 7. The excitation wavelength for fluorescence spectra was set at 347 nm, and the emission spectrum was recorded over the range of 360–550 nm.

### 2.3. Determination of Antioxidant Properties

Antioxidant activity was determined using established methods with modifications [16,17,18,19]. A 5 mg/mL sample solution was prepared and then diluted to five different concentrations (1, 2, 3, 4, and 5 mg/mL), mixed thoroughly, and set aside. Vitamin C at the same concentrations served as the positive control.

#### 2.3.1. DPPH Radical Scavenging Property

Take 2 mL of the sample solution and 2 mL of 0.2 mmol/L DPPH solution in anhydrous ethanol (1:1, *v*/*v*). Vortex the mixture and incubate it in the dark for 30 min, then measure the absorbance A_2_ at 517 nm.

DPPH radical scavenging activity (%) = [1 − (A_2_ − A_1_/A_0_)] × 100 (1)

where A_1_ is the absorbance of the sample group (sample solution plus DPPH solution); A_2_ is the absorbance of the sample background, measured with anhydrous ethanol instead of the DPPH solution; and A_3_ is the absorbance of the control, measured with distilled water instead of the sample solution.

#### 2.3.2. ABTS Radical Scavenging Property

Prepare ABTS working solution with an absorbance of 0.7 ± 0.02 at 734 nm. Add 0.5 mL of each sample to 2 mL of the ABTS working solution. Incubate the mixture at 4 °C for 10 min, then measure the absorbance at 734 nm

ABTS radical scavenging activity (%) = [1 − (A_2_ − A_1_/A_0_)] × 100 (2)

where A_1_ is the absorbance of the sample group; A_2_ is the absorbance of the sample background, measured with distilled water instead of the ABTS working solution; and A_3_ is the absorbance of the control, measured with distilled water instead of the sample solution.

#### 2.3.3. Hydroxyl Radical (·OH) Scavenging Property

Take 1 mL of the sample solution and 1 mL of FeSO_4_ solution (9 mmol/L); mix them, then add 1 mL of salicylic acid solution (9 mmol/L). After allowing the reaction to proceed at room temperature for 10 min, add 1 mL of H_2_O_2_ solution (8 mmol/L) to terminate the reaction. Subsequently, incubate the mixture in a dark water bath at 37 °C for 30 min, then measure the absorbance at 510 nm (A_2_).

·OH radical scavenging activity (%) = [1 − (A_2_ − A_1_/A_0_)] × 100 (3)

where A_1_ is the absorbance of the sample group; A_2_ is the absorbance of the sample background, measured with distilled water instead of the H_2_O_2_ solution; and A_3_ is the absorbance of the control, measured with distilled water instead of the sample solution.

#### 2.3.4. FRAP

Take 1 mL of the sample solution and add 2.5 mL of PBS (pH 6.6), followed by 2.5 mL of 1% K_4_Fe(CN)_6_. Incubate the mixture at 50 °C in a dark water bath for 20 min. After cooling, add 2.5 mL of 10% trichloroacetic acid to terminate the reaction. Centrifuge the solution at 4000 rpm for 10 min. Transfer 2.5 mL of the supernatant, then add 2.5 mL of ultrapure water and 0.5 mL of 0.5% FeCl_3_. Allow the reaction to proceed for 30 min, then measure the absorbance at 700 nm.

### 2.4. Determination of Hypoglycaemic Activities in Vitro

The α-amylase inhibitory activity assay was conducted using a method reported in the literature and modified in [20]. The procedure was as follows: Acarbose was used as the positive control, and the sample inhibition rate was determined via the DNS method. First, 200 μL of sample solution was mixed with 100 μL of 0.5 U/mL α-amylase solution and incubated at 37 °C for 20 min. Then, 200 μL of 2.0% soluble starch solution was added, followed by incubation at 37 °C for an additional 10 min. Next, 200 μL of DNS reagent was added, and the mixture was boiled for 5 min. After cooling and dilution with 5 mL of distilled water, the absorbance was measured at 540 nm. All measurements were performed in triplicate.

The α-glucosidase inhibitory activity assay was conducted using a modified method described in the literature [21]. The procedure was as follows: Acarbose served as the positive control. Solutions of 0.01 mol/L p-nitrophenyl-α-D-glucopyranoside (PNPG) and 0.1 U/mL α-glucosidase were prepared in 0.1 mol/L phosphate-buffered saline (PBS) at pH 6.6. For the sample group, 50 μL of sample solution and 50 μL of α-glucosidase solution were mixed and incubated at 37 °C for 10 min. Subsequently, 50 μL of PNPG solution was added, and the mixture was incubated at 37 °C for an additional 30 min. The reaction was terminated by adding 50 μL of 1.0 mol/L sodium carbonate solution. Absorbance was measured at 400 nm. In the control group, the sample solution was replaced with phosphate buffer. The blank group replaced both the enzyme and sample solutions with phosphate buffer, while the background group replaced only the enzyme solution with phosphate buffer. All measurements were performed in triplicate. The α-glucosidase inhibition rate was calculated using the following equation:

Inhibition rate (%) = [1 − (A_2_ − A_1_/A_0_)] × 100 (4)

where A_2_ is the absorbance of the sample, A_1_ is the absorbance of the control group, and A_0_ is the absorbance of the blank (with PBS solution replacing the sample solution).

### 2.5. Non-Targeted Metabolomics Analysis

#### 2.5.1. Metabolite Extraction

A 50 mg mass of each sample was added to 300 μL of 80% methanol containing three medium-sized steel balls. The tissue was homogenised and disrupted using a tissue homogeniser (JXFSTPRP-24, Jingxin, Shanghai, China), then 700 μL of 80% methanol was added. The mixture was vortexed, sonicated for 20 min in an ice bath, left to stand at –20 °C for 2 h, and then centrifuged at 16,000 rpm and 4 °C for 20 min. The supernatant was removed and evaporated to dryness using a high-speed vacuum centrifuge (Concentrator Plus, Eppendorf, Hamburg, Germany). For mass spectrometry analysis, the residue was resuspended in 100 μL of 50% methanol, then centrifuged at 20,000× *g* and 4 °C for 15 min; the supernatant was injected for analysis.

#### 2.5.2. UPLC-MS/MS Analysis

Throughout the analysis, samples were maintained at 4 °C within an autosampler. A SHIMADZU-LC30 ultra-high-performance liquid chromatography (UHPLC, Shimadzu Corporation, Kyoto, Japan) system was employed, utilising an ACQUITY UPLC^®^ HSS T3 column (2.1 × 100 mm, 1.8 µm) (Waters, Milford, MA, USA). The injection volume was 4 μL, with a column temperature of 40 °C and a flow rate of 0.3 mL/min. The chromatographic mobile phases comprised phase A, 0.1% formic acid in water, and phase B, acetonitrile. The gradient elution programme was as follows: 0–2 min, 0% B; 2–6 min, B increased linearly from 0% to 48%; 6–10 min, B increased linearly from 48% to 100%; 10–12 min, B held at 100%; 12–12.1 min, B decreased linearly from 100% to 0%; 12.1–15 min, B held at 0%. A method in the literature was referred to and modified accordingly [22].

### 2.6. Statistical Analysis

Each experiment was repeated three times. Data are presented as the mean ± standard deviation (SD). Graphs were plotted using Origin 2021 software, and data analysis was performed using SPSS 27.0.1 software.

## 3. Results

### 3.1. Electronic Tongue Analysis

**Figure 1:**
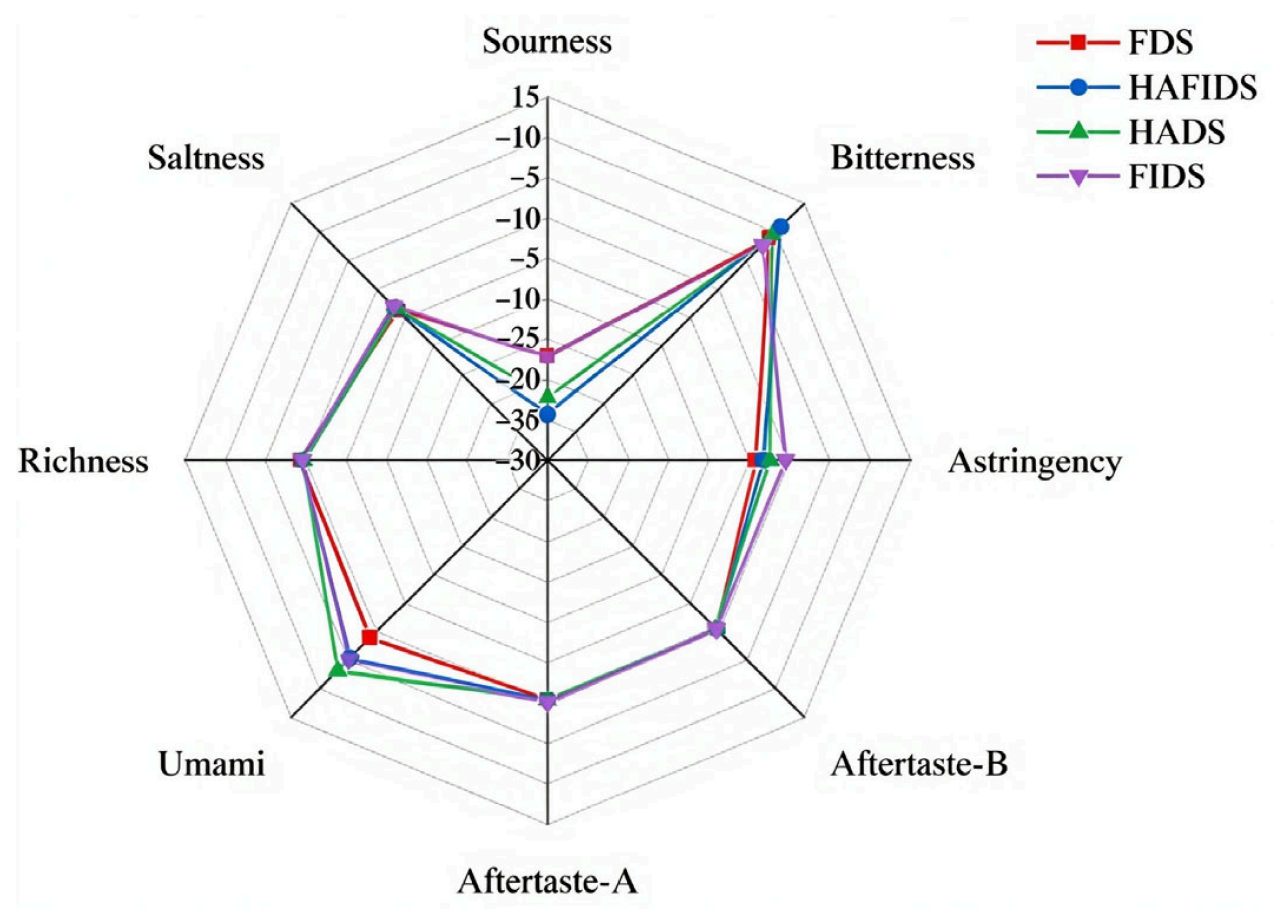
Electronic tongue radar diagram.

An electronic tongue is capable of detecting basic taste attributes, including sourness, astringency, bitterness, aftertaste-A (the duration of astringency in the mouth), aftertaste-B (the duration of bitterness in the mouth), umami, richness, and saltiness [23]. As shown in Figure 1, all samples scored negatively for sourness and saltiness, whilst scoring higher for bitterness. The results showed that the other three samples (HADS, FIDS, and HAFIDS) scored significantly higher than FDS in terms of bitterness, aftertaste-B, umami, and richness. In particular, aftertaste-B and umami were perceived most strongly in HADS, whilst saltiness was most pronounced in HAFIDS. Meanwhile, the strongest sourness, astringency, and aftertaste-A were perceived in FIDS. This is most likely due to protein hydrolysis during the high-temperature drying process, which promoted the release of small peptides and free amino acids, thereby significantly enhancing the soup’s umami, richness, and saltiness.

### 3.2. Ultraviolet Absorption Spectra Analysis

**Figure 2:**
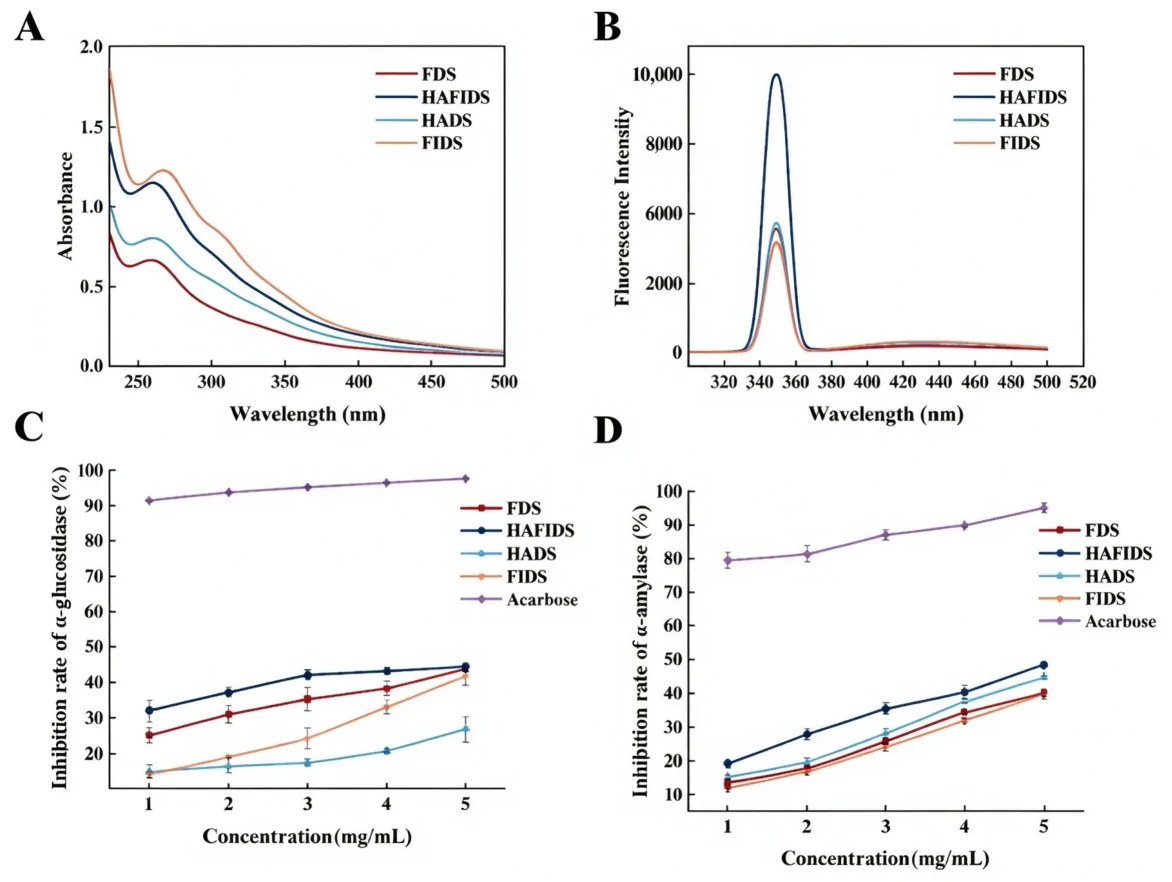
Ultraviolet absorption spectra, fluorescence spectroscopy results, and hypoglycaemic activity. (**A**): ultraviolet (UV) absorption spectra; (**B**): fluorescence spectra; (**C**): α-glucosidase inhibitory activity; (**D**): α-amylase inhibitory activity.

Ultraviolet absorption spectroscopy is frequently employed to detect the occurrence of the Maillard reaction [24]. A comparative examination of ultraviolet spectra was performed for all four samples across the wavelength interval spanning 200 to 500 nm. As shown in Figure 2A, a maximum absorption peak appeared at 275 nm. Significant variations were observed between 250 and 300 nm, likely indicating Maillard reactions occurring during drying and boiling processes, which resulted in an absorption peak at 280 nm. The absorbance measurements for HADS, HAFIDS, and FIDS were all higher than those for FDS. This disparity indicates that the thermal drying procedure induced structural modifications in the proteins or peptide fractions derived from *D. rubrovalvata*. Additionally, aromatic amino acids, including tryptophan and tyrosine, were detected, contributing to the increased ultraviolet absorbance values [25].

### 3.3. Fluorescence Spectroscopy Analysis

During the early phase of the Maillard reaction, fluorophores may emerge as intermediates prior to the development of coloured compounds. Accordingly, fluorescence spectroscopic analysis serves as a viable method for tracking the progression of this reaction [26]. Figure 2B illustrates the fluorescence spectral shifts observed across the four samples. The maximum fluorescence emission wavelength was recorded at 347 nm, with elevated fluorescence intensity detected between 300 and 360 nm following excitation at 350 nm. This indicates the formation of fluorescent compounds within *D. rubrovalvata* during processing. These compounds likely contain nitrogen or feature cyclic unsaturated carbon structures (e.g., polycyclic aromatic hydrocarbons or heterocyclic compounds) [25]. The ranking in terms of fluorescence intensity was HAFIDS > HADS > FDS > FIDS, suggesting that protein hydrolysis may induce structural alterations around aromatic amino acid residues [27]. Because of their distinctive conjugated structures and electronic transitions, these newly formed compounds exhibit red-shifted fluorescence with diminished intensity [28].

### 3.4. Hypoglycaemic Activity

α-Amylase and α-glucosidase are enzymes that participate in breaking down carbohydrates. The final products of starch hydrolysis by α-amylase are maltose, sucrose, and glucose. These products are further hydrolysed into monosaccharides by α-glucosidase. The inhibition of both enzymes is known to suppress an increase in blood glucose [29]. As their concentrations increased, so did the α-amylase and α-glucosidase inhibition rates of the four samples and acarbose. Acarbose exhibited the greatest inhibitory effect on α-amylase, followed by HAFIDS, HADS, FDS, and FIDS. Similarly, acarbose showed the strongest inhibition of α-glucosidase activity, followed by HAFIDS, FDS, FIDS, and HADS. These inhibitory effects are illustrated in Figure 2C,D. The observed differences may be attributed to changes in compounds—such as polysaccharides and phenolic compounds—resulting from the drying and steaming processes applied to *D. rubrovalvata*. Previous research indicates that these compounds influence inhibitory activity against both α-amylase and α-glucosidase [30].

### 3.5. Antioxidant Activity Analysis

**Figure 3:**
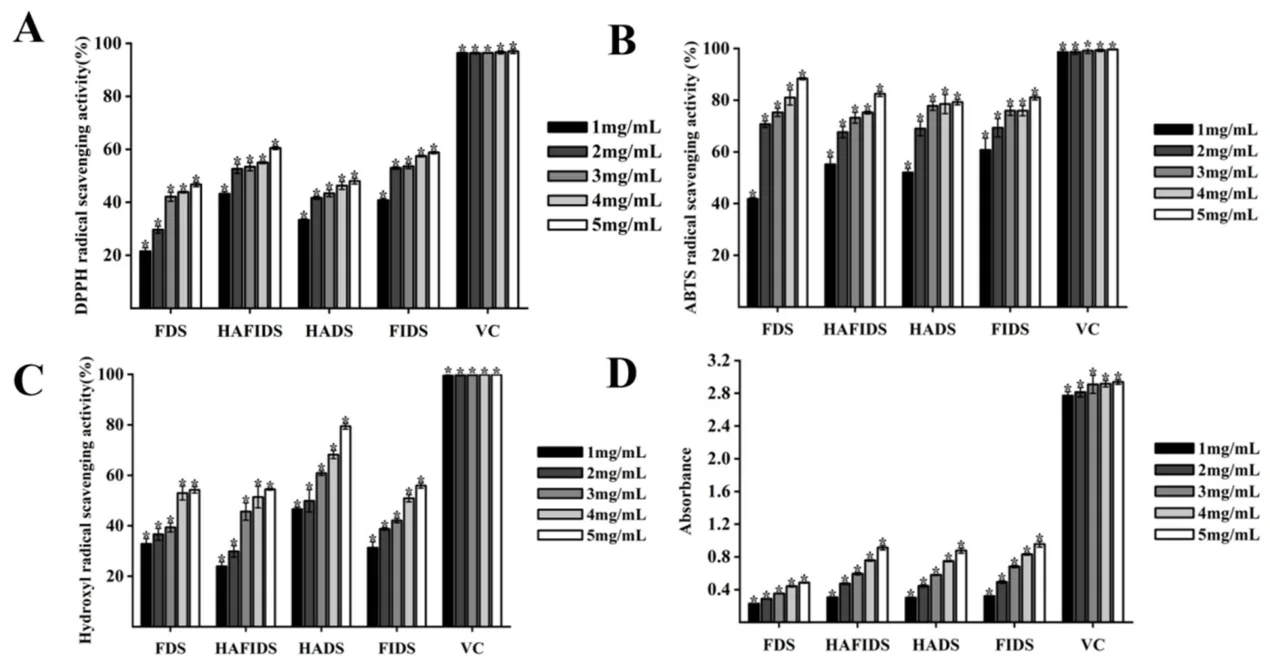
Antioxidant activity of soups. (**A**): DPPH· scavenging activity; (**B**): ABTS· scavenging activity; (**C**): ·OH scavenging activity; (**D**): FRAP (VC was used as a positive reference only). (* Indicates a statistically significant difference).

Active components in edible fungi, such as ergosterol, polysaccharides, and soluble sugars, exhibit antioxidant effects through mechanisms including free radical scavenging and metal ion chelation. Additionally, the Maillard reaction, induced by drying or heat treatment, further enhances their antioxidant activity [31]. As shown in Figure 3, the antioxidant potential of the four samples was determined using DPPH·, ABTS·, and OH· radical scavenging assays, in addition to the FRAP test. Each of the four samples showed notable DPPH·, ABTS·, and OH· radical scavenging activity, which increased markedly with concentration. The order of DPPH scavenging capacity was Vc > HAFIDS > FIDS > FDS > HADS, while the ABTS scavenging ability was ranked as follows: Vc > FDS > HAFIDS > FIDS > HADS. The ·OH scavenging ability was ranked Vc > HADS > FIDS > HAFIDS > FDS, and the ferric-reducing power followed the sequence Vc > HAFIDS > FIDS > HADS > FDS. The high temperatures and brief cooking times likely contributed to this enhanced antioxidant capacity. Under these conditions, active polysaccharides and proteins were released into the soup. As a result, all four samples showed concentration-dependent increases in scavenging activity. This phenomenon can likely be attributed to thermal treatment. Proteins, fats, and carbohydrates all degrade during drying and cooking. This process liberates reducing sugars and amino acids, and interactions among these released compounds then take place [32].

### 3.6. Metabolomic Analysis

#### 3.6.1. Overview of Metabolites

**Figure 4:**
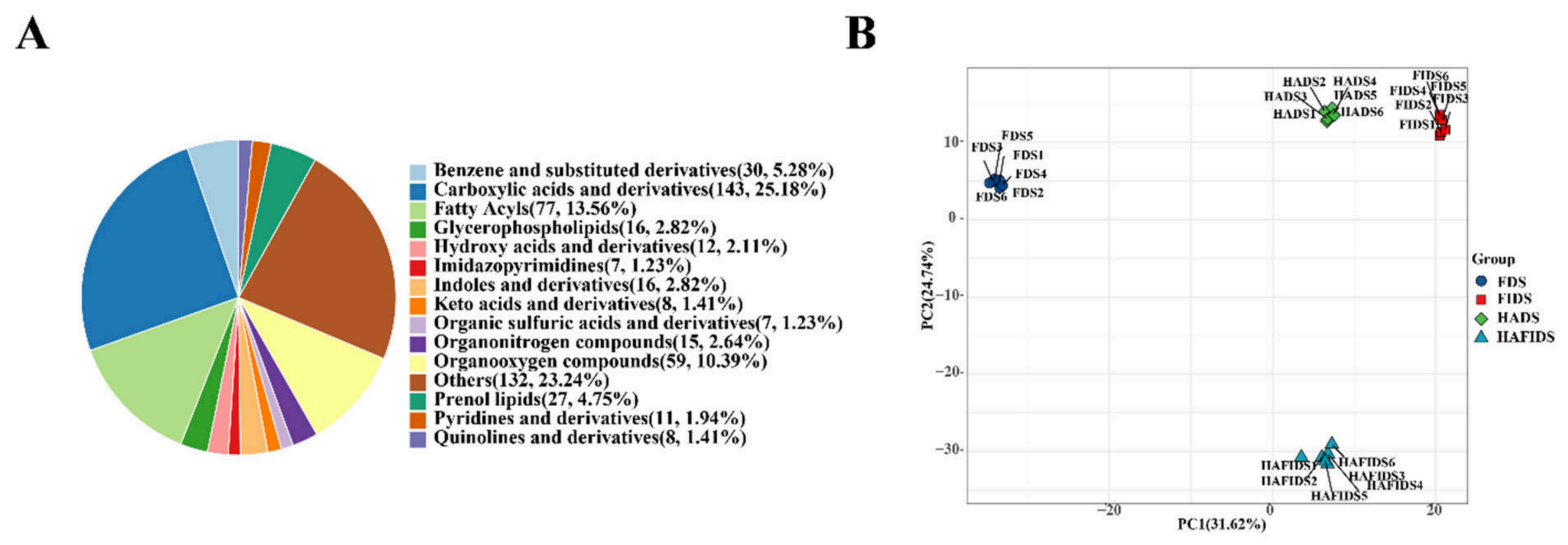
Metabolites in mushroom soups made from *D. rubrovalvata* dried using different methods. (**A**): Pie chart of biochemical categories of 716 non-VOCs. (**B**): PCA score plot.

Using LC-MS/MS non-targeted metabolomics, changes in key metabolites within the *D. rubrovalvata* soups (FDS, HAFIDS, HADS, and FIDS) were revealed. Table S1 displays the 716 identified metabolites. In the PCA plot, replicates clustered tightly within each group, while the four sample groups were clearly differentiated from each other (Figure 4B). This indicated that the metabolite profiles of the D. rubrovalvata soups differed among the four drying processes. Along PC1 (31.62%), FDS, HAFIDS, HADS, and FIDS were clearly distinguishable. Furthermore, HADS was markedly separated from FIDS and FDS along PC2 (24.74%).

#### 3.6.2. Multivariate Analysis

To investigate metabolic differences among FDS, HAFIDS, HADS, and FIDS, pairwise comparisons of metabolite abundances were conducted across the four sample groups.

**Figure 5:**
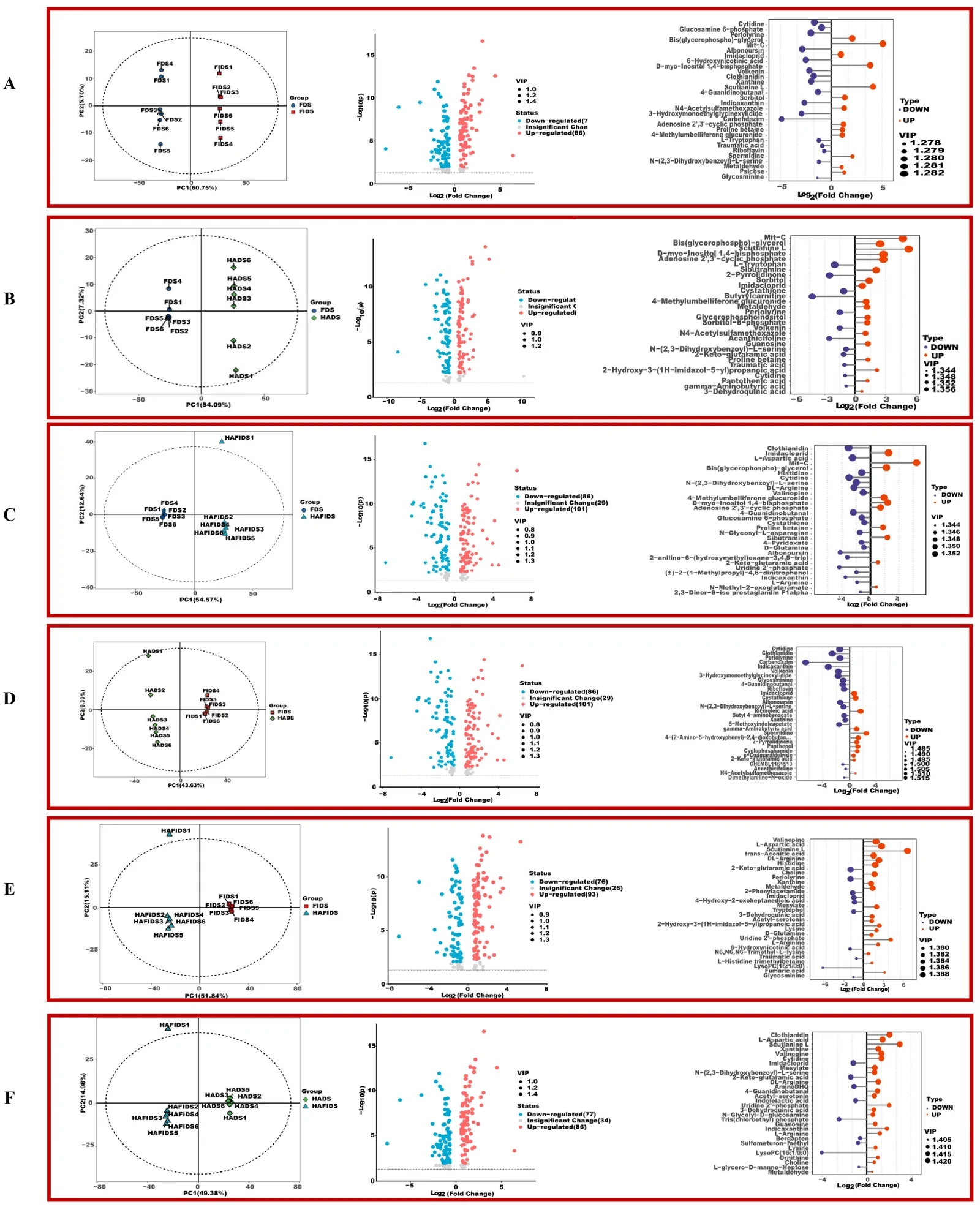
Metabolite differential analysis in pairwise comparisons across four groups of mushroom soups made from *D. rubrovalvata* dried using different methods, including top 30 metabolites with highest VIP scores, fold change (FC) bar charts, and volcano plots of the different non-VOCs for each pairwise comparison. (**A**): FDS vs. FIDS; (**B**): FDS vs. HADS; (**C**): FDS vs. HAFIDS; (**D**): HADS vs. FIDS; (**E**): HAFIDS vs. FIDS; (**F**): HAFIDS vs. HADS.

As shown in Figure 5, the comparisons between FDS and FIDS, FDS and HADS, FDS and HAFIDS, HADS and FIDS, HAFIDS and FIDS, and HAFIDS and HADS identified 234, 200, 216, 115, 194, and 197 differentially abundant metabolites (DAMs), respectively. Within FIDS, 127, 63, and 105 DAMs exhibited higher abundances compared to those detected in FDS, HADS, and HAFIDS, respectively. These results indicate that FID could be a successful approach for producing a large quantity of metabolites.

The top 30 DAMs were selected as biomarkers for mushroom soups made from *D. rubrovalvata* dried using different methods. Compared to FDS, FIDS exhibited elevated levels of 127 metabolites, HADS showed increased levels of 101 metabolites, and HAFIDS demonstrated higher levels of 115 metabolites. This difference could be due to the oxidation and breakdown of certain compounds when exposed to high-temperature drying, resulting in the formation of novel metabolites [33]. Cytidine and N-(2,3-dihydroxybenzoyl)-L-serine were consistently downregulated in HADS, HAFIDS, and FIDS relative to FDS, indicating that these metabolites were present in the highest concentrations in FDS and defined its distinctive metabolic profile. In contrast to FDS, HADS, HAFIDS, and FIDS exhibited a greater number of upregulated non-VOCs. This phenomenon could be due to a greater extent of heat-induced chemical transformation and metabolite release in *D. rubrovalvata* soup when subjected to HAD and FID conditions [34].

The number of metabolites differing between the HADS and FIDS groups was significantly lower than those found in the other group comparisons. A total of 115 non-VOCs were identified between HADS and FIDS, with 65 exhibiting higher concentrations in FIDS and 52 showing lower concentrations. Among the top 30 differing metabolites between HADS and FIDS, perlolyrine and carbendazim were found at elevated levels. Aspartic acid, histidine, and lysine were more abundant in FIDS than in HAFIDS, while xanthine and riboflavin were more abundant in HADS than in FIDS. Adenosine 2′,3′-cyclic phosphate and LysoPC (16:1/0:0) were more abundant in HAFIDS. These compounds play significant roles in DNA and RNA structures. These results provide a clear understanding of the distinct metabolites and their biomarkers across various drying methods. The varying accumulation and degradation of metabolites in the mushroom soups made from *D. rubrovalvata* dried using different methods may have been influenced by factors such as temperature, enzyme activity, and drying duration [35].

#### 3.6.3. K-Means Clustering Analysis for Different Metabolites

**Figure 6:**
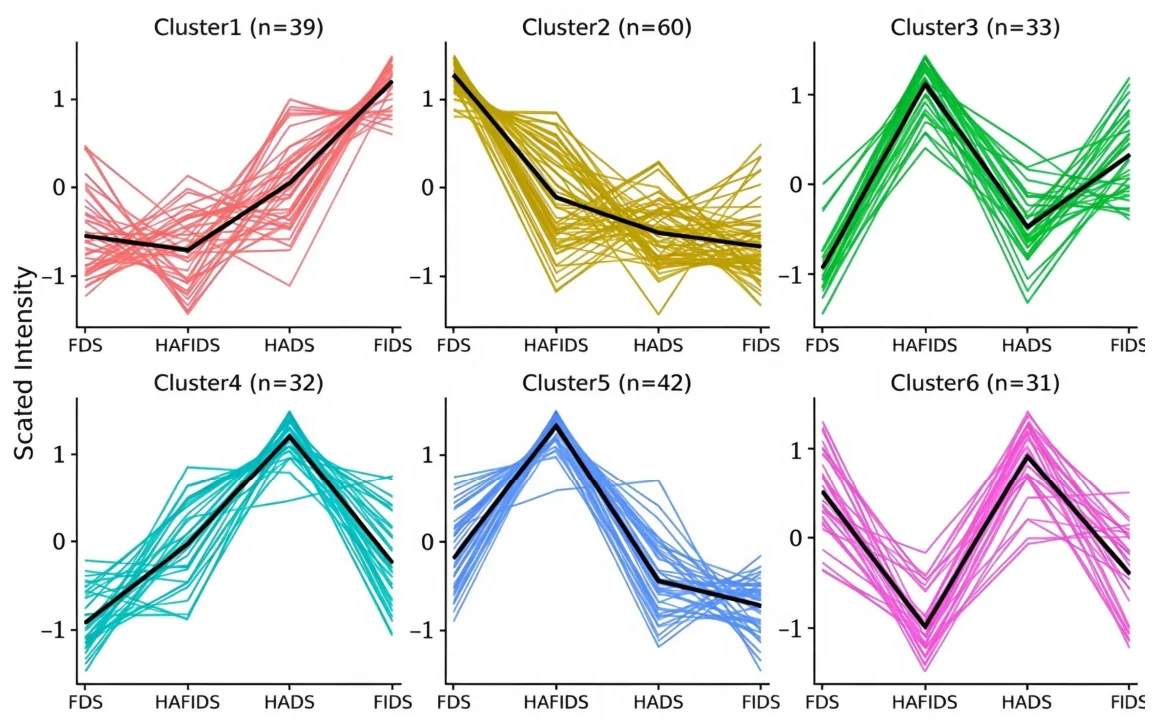
K-means clustering analysis of the expression profiles of various non-volatile organic compounds (non-VOCs) in mushroom soups made from *D. rubrovalvata* dried using different methods. The *y*-axis represents the standardised content of each metabolite, and the *x*-axis represents different samples.

Differences in the relative concentrations of non-VOCs across the groups were evaluated. All distinct non-VOC data were normalised and subsequently subjected to K-means clustering analysis [36]. As shown in Figure 6, using the K-means method, 238 distinct non-VOCs were classified into six subcategories. Subcategory 2 contained 60 non-VOCs, with FDS levels higher than those in the other three categories. This suggests that high temperatures or prolonged drying times may degrade such metabolites, whereas FD effectively preserves them. Subcategory 2 comprised sugars and alcohols, including D-glucose, sorbitol, and allulose. In subclasses 3 and 5, HAFIDS exhibited 33 and 42 non-VOCs, respectively, at levels surpassing those in the other three subclasses. These included amino acids and derivatives (e.g., methionine, leucine, L-aspartic acid), nucleotides (e.g., cytidine, uridine), and sugars/derivatives (e.g., N-hydroxyacetyl-D-glucosamine), indicating that processing effectively preserved nutritional components and metabolic accumulation. Subclasses 4 and 6 contained 32 and 31 non-VOCs, respectively, at levels higher in HADS than in the other three subclasses. HADS exhibited elevated levels of epoxy fatty acids, oxygenated fatty acids, and amino acid degradation products (e.g., 2-pyrrolidone, benzaldehyde). This suggests that high-temperature conditions during hot-air drying and boiling accelerated the oxidation and degradation of lipids and amino acids [37].

#### 3.6.4. Key Metabolic Pathways

**Figure 7:**
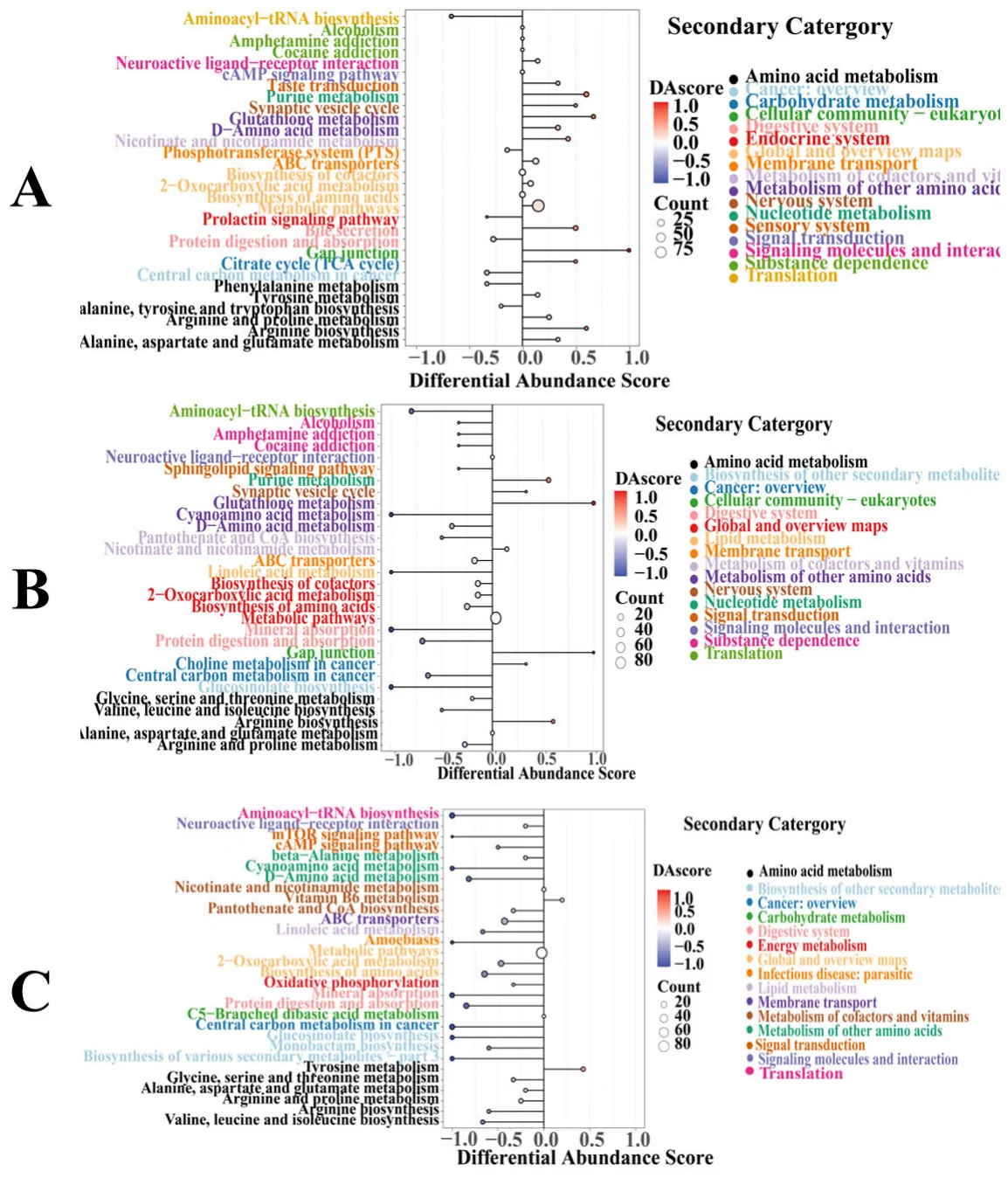

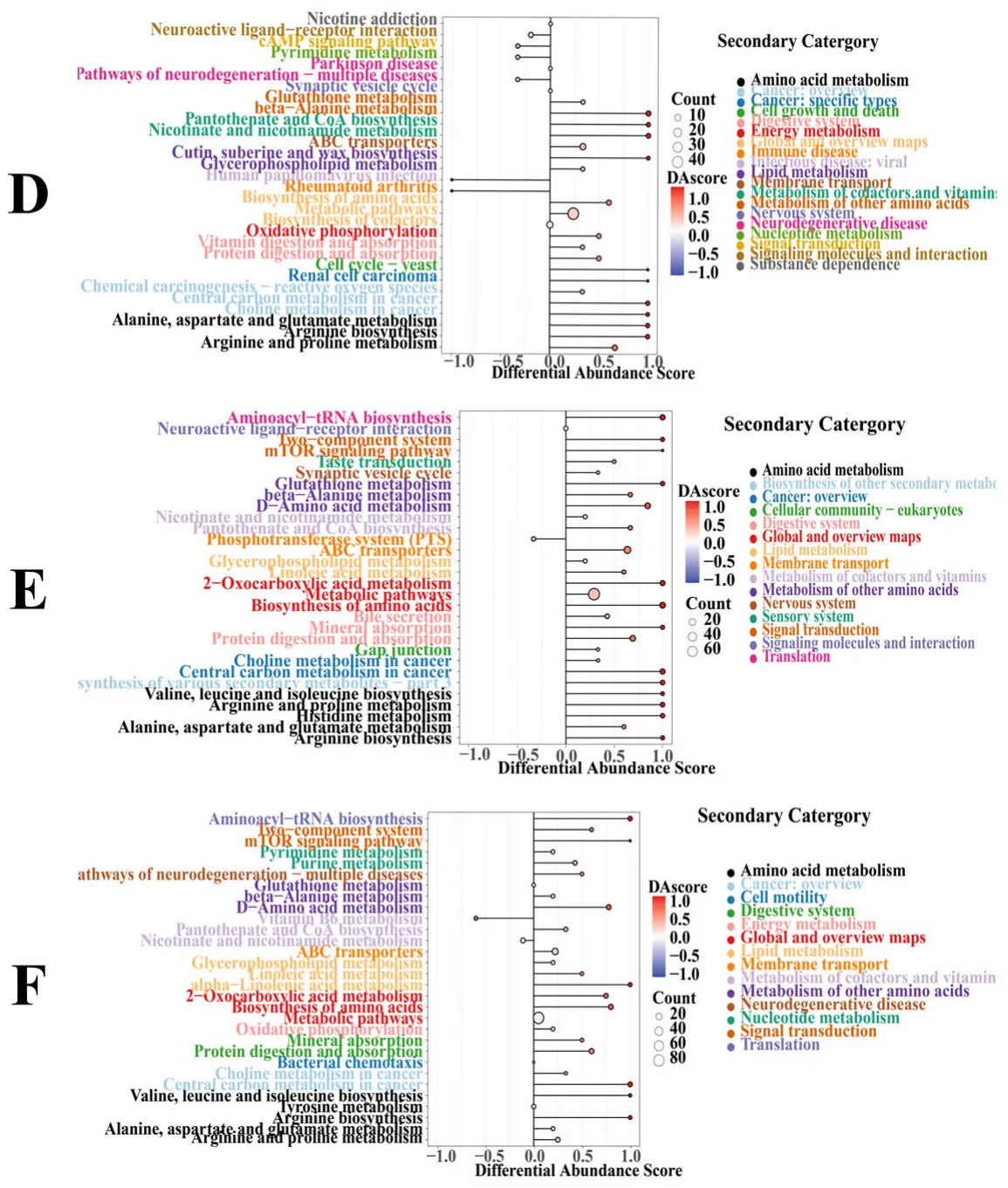
Key metabolic pathways of mushroom soups made from *D. rubrovalvata* dried using different methods. (**A**): FDS vs. FIDS; (**B**): FDS vs. HADS; (**C**): FDS vs. HAFIDS; (**D**): HADS vs. FIDS; (**E**): HAFIDS vs. FIDS; (**F**): HAFIDS vs. HADS.

The main metabolic pathways linked to metabolites that showed significant differences in expression among different *D. rubrovalvata* soups were examined. In Figure 7, the horizontal axis represents the DA score, which reflects the overall trend of change across all metabolites within a metabolic pathway; the vertical axis denotes the pathway, with different colours indicating distinct classifications. Each circle corresponds to a pathway, with the circle size indicating the number of annotated metabolites within that pathway. Colours range from blue to red, representing DA scores from −1 to 1. A DA score of −1 indicates a decrease in abundance for all metabolites within the pathway, while a score of 1 indicates an increase. Values closer to 1 or −1 signify a stronger overall tendency toward upregulation or downregulation within the pathway. The differentially expressed metabolites identified in the *D. rubrovalvata* soups primarily relate to seven key metabolic pathways: amino acid metabolism, other amino acid metabolism, nucleotide metabolism, translation, carbohydrate metabolism, and lipid metabolism. The amino acid metabolism pathway consistently emerged as the dominant pathway in all comparisons involving *D. rubrovalvata* soups. These included the biosynthesis of arginine; the metabolism of alanine, aspartic acid, and glutamic acid; the metabolism of arginine and proline; the biosynthesis of phenylalanine, tyrosine, and tryptophan; phenylalanine metabolism; and tyrosine metabolism. All these pathways showed a greater number of annotated differential metabolites, leading to a broader range of metabolites. This conclusion is based on a KEGG-based metabolite annotation and set enrichment analysis. Consequently, it is hypothesised that amino-acid-related metabolites showed the most pronounced differences in relative abundance among the four soups during both the drying and cooking processes of *D. rubrovalvata* soup. Different drying methods for edible fungi also reveal significant variations in amino acid metabolism [38]. Heat treatment generates oxidative products by modifying the side chains of amino acids, resulting in alterations in amino acid metabolism [39]. Therefore, it is hypothesised that the metabolism of amino acids in *D. rubrovalvata* soup is affected by the temperature and the rate of heating during drying. Overall, amino acids, sugar alcohols, and organic acids, along with their derivatives, were identified as the primary biomarkers distinguishing different drying methods, with their associated metabolic pathways exhibiting the highest activity. These compounds constitute the key sources of flavour characteristics [36].

#### 3.6.5. Differences in Flavour-Defining Compounds

**Figure 8:**
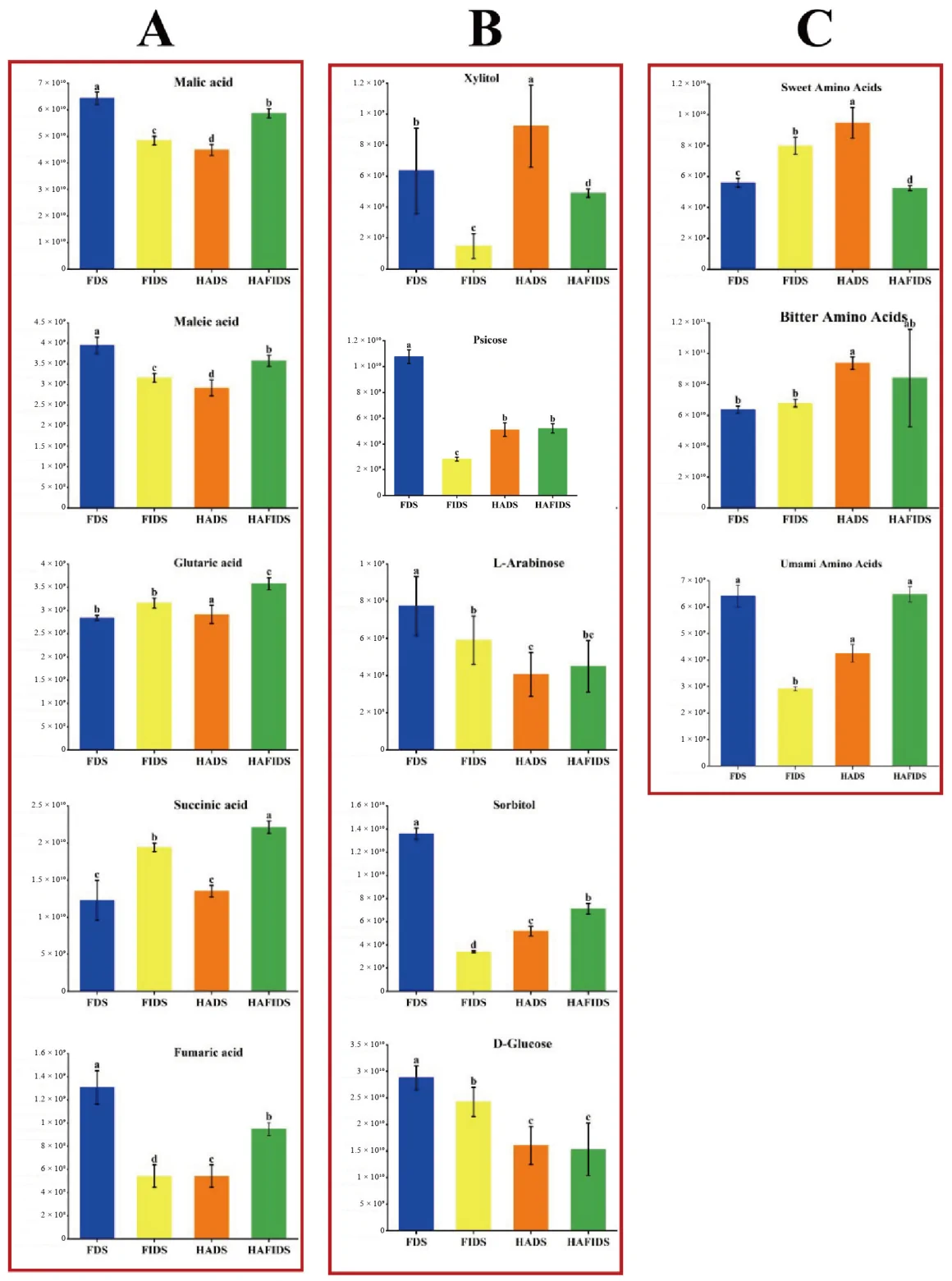
Changes in organic acid (**A**), sugar alcohol (**B**), and free amino acid (**C**) contents in mushroom soups made from *D. rubrovalvata* dried using different methods. Note: Significant differences between groups (*p* < 0.05) are denoted by different letters (a–d); different letters above bars indicate significant differences.

The primary components that influence the taste of edible mushrooms are free amino acids, organic acids, and soluble sugar alcohols [23]. Metabolomic analysis identified 12 potential taste-related amino acids, including two umami amino acids (aspartic acid and glutamic acid), four sweet amino acids (proline, alanine, serine, and threonine), and six bitter amino acids (leucine, histidine, phenylalanine, arginine, lysine, and methionine) [40]. As shown in Figure 8, bitter amino acids were the most prevalent and abundant, exhibiting significantly higher levels in HADS compared to the other three soups. The abundance of umami amino acids was greater in HAFIDS than in FDS, while HADS levels fell between those of FDS and HAFIDS, with no significant difference observed. For the sweet amino acids, HADS exhibited significantly higher abundance than the other three soups. Compared to other drying techniques, FD potentially increases the umami amino acid content in *D. rubrovalvata* soup while reducing bitter amino acid levels. Glycine and threonine are characteristic sweet amino acids, and their derivatives are considered typical compounds accountable for the inherent sweetness found in mushrooms [41].

Organic acids play a pivotal role in flavour characterisation. Among the 37 organic acids analysed, we focused on five key compounds: succinic acid, fumaric acid, malic acid, citric acid, and succinic acid. No significant differences were observed between FDS and HAFIDS, although HAFIDS exhibited the highest total abundance of seven organic acids, followed by FDS and FIDS, with HADS showing the lowest levels. The differences in malic acid abundance among the four soups were not significant. HADS exhibited the highest citric acid abundance, followed by FDS and FIDS, with that for FDS being the lowest; HAFIDS exhibited markedly higher succinic acid abundance than the other three soups. These three organic acids, present in high amounts, are all important products of the tricarboxylic acid cycle. The organic acid contents in the other three soups were higher than those in FDS. Notably, the low citric acid content in FDS was primarily due to anaerobic conditions during FD, which impeded citric acid production [42].

Sugars and alcohols are the primary contributors to the sweetness of the *D. rubrovalvata* soup. We focused our research on five significant sugar alcohols: glucose, arabinose, sucrose, sorbitol, and xylitol. Regarding these five sugar alcohols, FDS exhibited a higher total sugar abundance than the others. Compared to the other three soups, FIDS contained significantly lower levels of arabinose, sucrose, sorbitol, and xylitol. One possible explanation for this is that these monosaccharides and alcohols participate in the Maillard reaction, leading to their substantial consumption during the FID period [43].

The aforementioned findings indicate that heating significantly influenced the abundance of flavour compounds, thereby enhancing the umami profile of *D. rubrovalvata* soup. During heating, protein degradation and nucleic acid breakdown generate freshness, sweetness, and bitterness; the tricarboxylic acid cycle produces organic acids that contribute acidity under aerobic conditions, while polysaccharide degradation and oxidation-derived sugars provide sweetness. This indicates that the overall sensory experience of the material is ultimately affected by taste generation, potentially involving additional metabolites. Differences in non-volatile organic compounds may explain the flavour variations in *D. rubrovalvata* soup prepared via different drying methods. Major contributors to flavour are likely found among several metabolite categories: lipids, amino acids and their derivatives, sugars and alcohols, nucleotides and their derivatives, and organic acids.

#### 3.6.6. Metabolic Pathway Network

This study sought to elucidate how drying methods affect the metabolite composition of *D. rubrovalvata* soup. Metabolic characteristics were analysed accordingly, employing KEGG network enrichment. Based on the obtained metabolomics data, the construction of a metabolic network map revealed that the metabolic processes of *D. rubrovalvata* soup primarily involve three core pathways: the tricarboxylic acid (TCA) cycle, amino acid metabolism, and nucleotide metabolism. It should be noted that the metabolites presented in this network originate from untargeted metabolomics showing only changes in relative abundance. Linkages between metabolites correspond to biochemical relationships annotated within KEGG and do not reflect experimentally measured metabolic fluxes.

**Figure 9:**
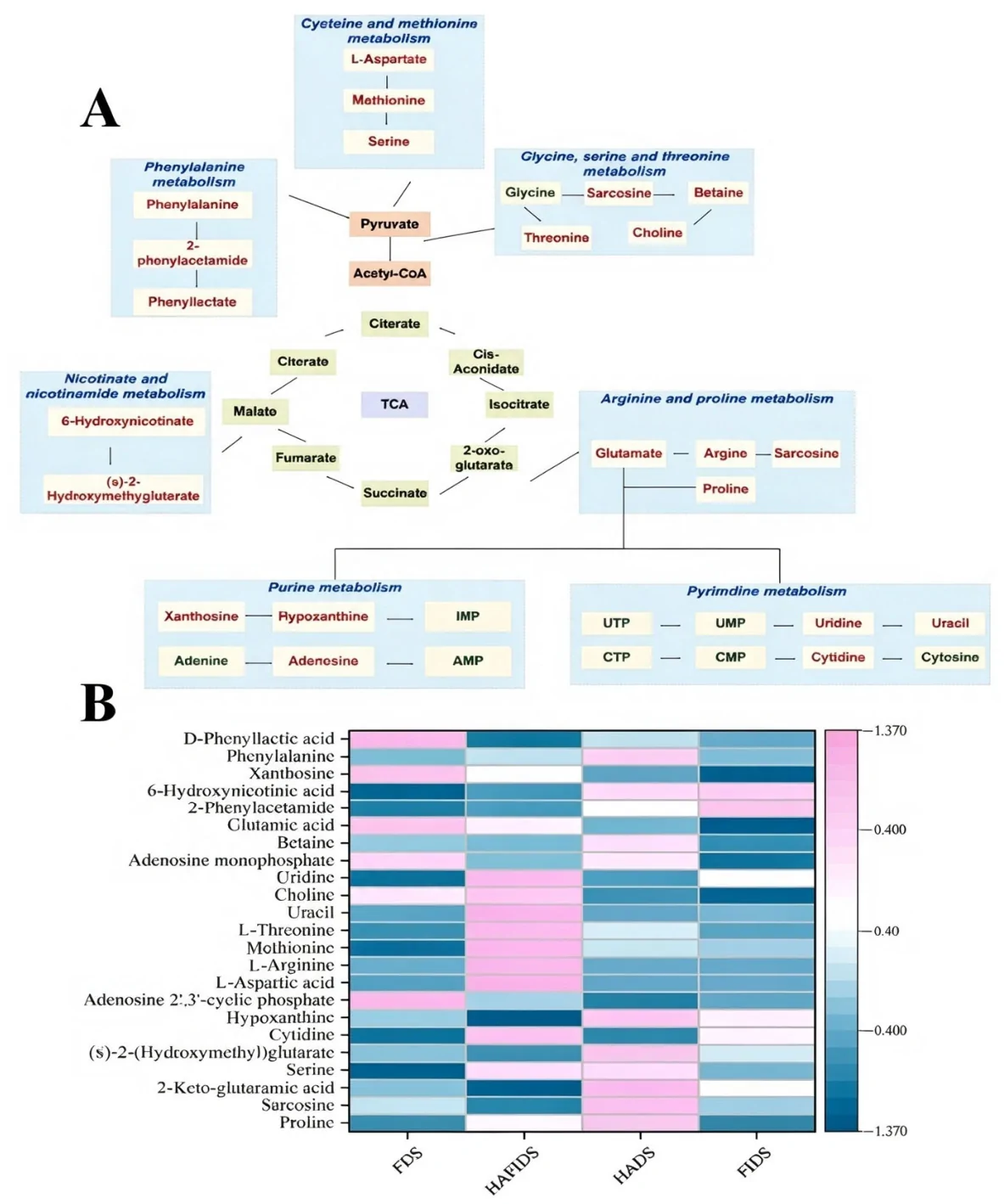
The metabolic pathway network of taste compounds (**A**) and key intermediates within the metabolic pathways (**B**) in mushroom soups made from *D. rubrovalvata* dried using different methods. Nodes correspond to metabolites identified via non-targeted metabolomics; node colour and size denote relative abundance across the four sample groups. Edges indicate biochemical relationships from the KEGG database. This network illustrates measured metabolite variation and documented biochemical connections; it provides no evidence for metabolic flux or pathway activation.

As shown in Figure 9, the TCA cycle serves as the central hub connecting various metabolic pathways, generating key intermediates such as succinate, fumarate, and malate. These intermediates act as essential precursors for the synthesis of important amino acids (glutamate, aspartate, alanine) and nucleotides (AMP, IMP). Pyruvate, a central metabolic intermediate, serves as the key entry point for the TCA cycle and is extensively involved in multiple pathways, including alanine, aspartate, and glutamate metabolism; cystine and methionine metabolism; glycine, serine, and threonine metabolism; phenylalanine metabolism; and arginine and proline metabolism [44]. Moreover, 2-oxoglutarate produced in the TCA cycle may further participate in glutamate synthesis. As a crucial downstream metabolic hub, glutamate connects the purine and pyrimidine metabolic pathways, and its metabolic alterations may lead to fluctuations in uracil, xanthine, and adenine [45].

## 4. Discussion

Differences in flavour compounds and bioactive substances among samples from the four drying processes were associated with four core pathways: amino acid metabolism, TCA cycle, carbohydrate metabolism, and nucleotide metabolism. This study highlights that these quality changes require nuanced interpretation.

The UV and fluorescence spectra reflect distinct Maillard reaction stages. Higher UV absorbance in all heat-dried soups indicates conjugated MRP accumulation. The fluorescence order (HAFIDS > HADS > FDS > FIDS) suggests that two-stage HAFID heating retains intermediate fluorescent MRPs, while prolonged far-infrared heating during the FID process promotes non-fluorescent melanoidin formation. The lower amounts of protein-derived fluorophores in FIDS may also account for this trend, marking a spectral analysis limitation.

Taste metabolites exhibit evident processing dependency. Thermal drying increases umami and bitter amino acid contents via protein hydrolysis and side-chain modification but depletes intrinsic sugars and sugar alcohols, with HAD additionally generating oxidative off-flavours. By contrast, FD preserves natural sweetness yet reduces umami intensity, which aligns with findings in dried mushrooms. Thus, HAFID’s superiority is specific to umami-oriented products rather than universally applicable.

HAFIDS ranked first among the four samples in terms of DPPH radical scavenging, FRAP, α-amylase inhibition, and α-glucosidase inhibition. Its lower ABTS and hydroxyl radical scavenging activities (where FDS and HADS performed best, respectively) likely reflect the different reaction mechanisms of these assays and the differential contributions of Maillard reaction products and endogenous phenolics to each antioxidant system.

The pathway enrichment results indicate correlational rather than causal metabolic variations. Elevated levels of TCA intermediates in dried samples likely result from cell rupture and metabolite release, rather than active metabolic flux. The dominant amino acid metabolism enrichment may have been biased by KEGG database annotation.

This study has several key limitations. Because the temperature and drying duration differed among the four processes, the effects observed here represent those of the overall drying process rather than those of any single parameter; dedicated single-factor designs would be needed to separate their individual contributions. Non-targeted metabolomics provides only relative quantification, precluding taste activity value calculations, and volatile compounds were not analysed. Future work combining targeted quantification, volatile metabolomics, simulated digestion, and in vivo validation will help refine this framework and support the standardised development of *D. rubrovalvata* soup products.

## 5. Conclusions

All four drying methods altered the Maillard reaction intensity, flavour characteristics, bioactivity, and metabolic profile composition of *D. rubrovalvata* soup. HAFIDS showed the highest DPPH radical scavenging activity, ferric-reducing power, and α-amylase and α-glucosidase inhibitory activity, together with the most pronounced enrichment of umami-related metabolites; FDS demonstrated the best retention of natural sweet-tasting compounds such as sugars and sugar alcohols, and the lowest degree of Maillard reaction. Differences in flavour compounds and bioactive substances among samples from the four drying processes were associated with four core pathways—amino acid metabolism, the tricarboxylic acid cycle, carbohydrate metabolism, and nucleotide metabolism—corresponding to distinct gustatory characteristics and levels of bioactivity in the *D. rubrovalvata* soups. This study provides insight into the metabolic basis of the differences in gustatory quality and bioactivity of *D. rubrovalvata* soups associated with the four drying processes. It provides a theoretical basis and technical support for the deep processing of *D. rubrovalvata* fungus, as well as for the flavour optimisation and quality control of edible fungus soups. Future work will combine omics-based analyses to further elucidate the synergistic mechanisms of key flavour and bioactive compounds, thereby advancing the development of high-value, standardised *D. rubrovalvata* soup products.

## Data Availability

The original contributions presented in this study are included in the article/Supplementary Materials. Further inquiries can be directed to the corresponding authors.

## Supplementary Materials

The following supporting information can be downloaded at: https://www.mdpi.com/article/10.3390/foods15213930/s1, Figure S1: Photographs of *D. rubrovalvata* ‘Wansun 2’ showing diagnostic morphological features (dark olive-green gleba-covered pileus, white reticulate indusium, and pinkish volva); Table S1: The identified metabolites in *Dictyophora rubrovalvata* soup; Table S2: Summary of the ITS-based molecular identification of the mushroom material. Reference [46] is cited in the supplementary materials.
