# Supplementary material for: Study on the Effects of Different Drying Methods on Nutritional Quality of Dictyophora rubrovalvata Soup Based on Metabolomics
Source: Foods. 2026 Nov 2;15(21):3930. doi: 10.3390/foods15213930 (PMC13762496; doi:10.3390/foods15213930)
Supplement: Supplementary file 1 [file foods-15-03930-s001.zip › foods-4582193-supplementary.pdf]

Supplementary Material

Manuscript ID: foods-4582193 — Study on the Effects of Different Drying Methods on Nutritional Quality of *Dictyophora rubrovalvata* Soup Based on Metabolomics

Statement on species identification

The mushroom material used in this study was the fruiting bodies of *Dictyophora rubrovalvata* (also spelled *D. rubrovolvata* in taxonomic databases such as NCBI Taxonomy and GBIF) cultivar 'Wansun 2' ('皖荪 2 号'). The identification of the material was confirmed by three independent lines of evidence: (1) morphological examination of fresh fruiting bodies at the cultivation facility; (2) molecular identification of the strain by ITS sequencing performed by an independent third-party laboratory; and (3) the documented pedigree of the registered cultivar 'Wansun 2', which was domesticated from a wild D. rubrovalvata strain collected in Anlong County, Guizhou Province, China, and officially registered by the Anhui Provincial Registration Committee for Non-major Crop Varieties in September 2021 (Certificate No. 皖品鉴登字第 2112005).

1. Photographic evidence (Supplementary Figure S1)

Photographs of the mushroom material were taken at the factory cultivation facility where the experimental material was produced. The fresh fruiting body displays the diagnostic morphological features of *D. rubrovalvata*: a conical pileus covered with dark olive-green mucilaginous gleba, a well-developed white reticulate indusium (skirt), a white hollow spongy stipe, and—most characteristically—a pinkish-red volva and pinkish fruiting-body "eggs" (the red volva is the defining trait of D. rubrovalvata, i.e., "红托竹荪").

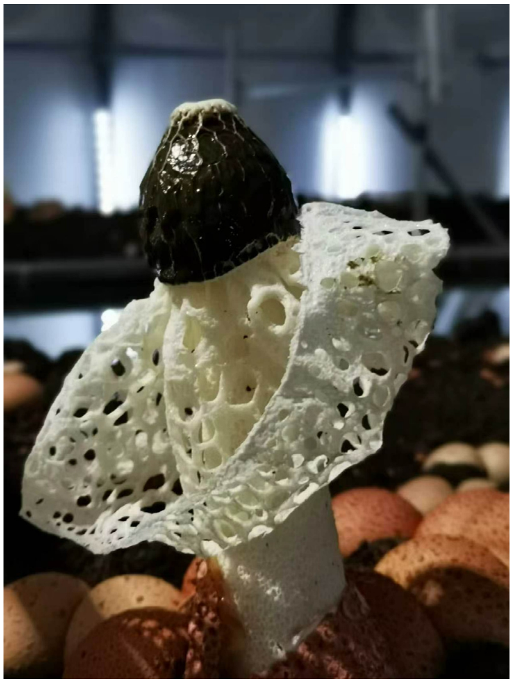

Figure S1a. Fresh fruiting body of *D. rubrovalvata* ‘Wansun 2’ showing the diagnostic dark olive-green gleba-covered pileus, white reticulate indusium, and pinkish volva at the base (pinkish “eggs” visible in the background).

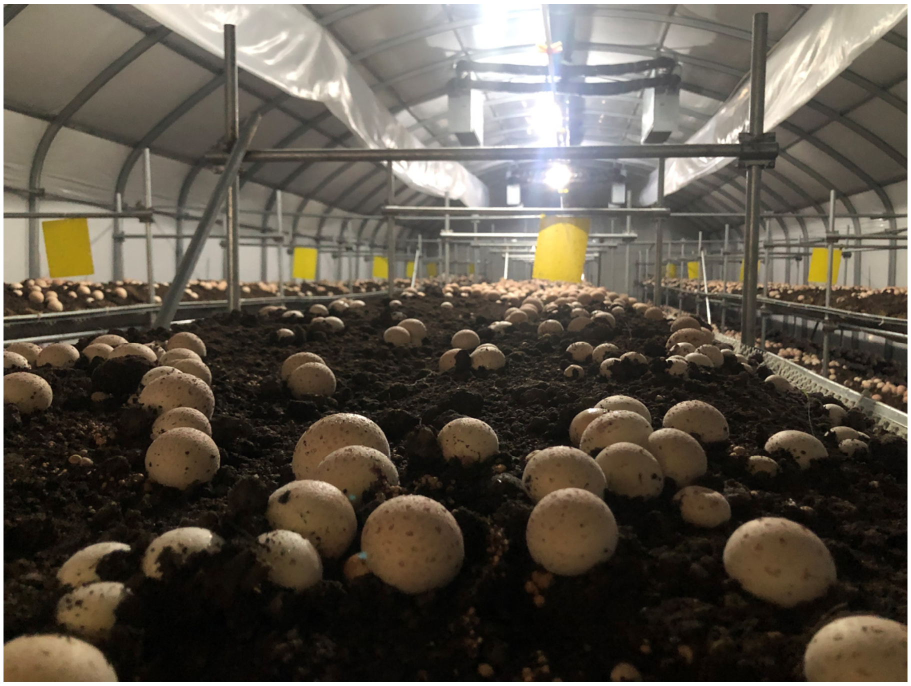

Figure S1b. Fruiting-body “eggs” of ‘Wansun 2’ under factory cultivation at the facility that supplied the experimental material.

2. Molecular evidence: ITS sequencing (Supplementary Table S2)

Molecular identification was performed by an independent third-party laboratory (Hefei Fengzhi Biotechnology Co., Ltd., Hefei, China; report dated 31 May 2022) commissioned by the Sericultural Research Institute, Anhui Academy of Agricultural Sciences. The ITS region was amplified with the universal primers ITS3 (5′-GCATCGATGAAGAACGCAGC-3′) and ITS4 (5′-TCCTCCGCTTATTGATATGC-3′), and the amplicon was Sanger-sequenced in both directions on an ABI 3730XL DNA Analyzer. BLAST analysis of the consensus sequence against the NCBI nucleotide database returned *D. rubrovolvata* as the top hit (Table S2).

Table S2. Summary of the ITS-based molecular identification of the mushroom material.

Item	Description
**Method**	PCR amplification of the ITS region (primers ITS3/ITS4) followed by bidirectional Sanger sequencing (ABI 3730XL)
**Top BLAST hit**	*Dictyophora rubrovolvata* (= *D. rubrovalvata*)
**Sequence identity**	99.61–100% (three samples of the ‘Wansun 2’ strain)
**Report**	Third-party identification report, Hefei Fengzhi Biotechnology Co., Ltd., 31 May 2022

3. Taxon-specific records: cultivar registration and published cultivar description

The cultivar ‘Wansun 2’ was bred by the Sericultural Research Institute, Anhui Academy of Agricultural Sciences, by systematic selection from a wild D. rubrovalvata strain collected in Anlong County, Guizhou Province, China. It was officially approved and registered by the Anhui Provincial Registration Committee for Non-major Crop Varieties in September 2021 (Certificate No. 皖品鉴登字第 2112005; scanned certificate provided as a separate PDF). A formal cultivar description, including morphological characteristics and cultivation traits, has been published:

Zhang, L.; Zhang, L.; Xin, L.; Chen, M.; Zhu, S.; Zhang, Y.; Wang, Y.; Bao, X. A New *Dictyophora rubrovalvata* Cultivar 'Wansun 2'. Acta Horticulturae Sinica 2023, 50 (S1), 93–94. https://link.cnki.net/doi/10.16420/j.issn.0513-353x.2022-1080
